# The Mechanism of Exophthalmos

**Published:** 1904-11-19

**Authors:** 


### THE MECHANISM OF EXOPHTHALMOS.

An experimental inquiry into the production of exophthalmos has recently been carried out by Dr. W. G. MacCallum and Dr. W. B. Cornell. It will be obvious that there are many kinds of exophthalmos which need no experimental elucidation, such, for example, as those which result from hæmorrhage, emphysema, exostoses, tumours, or cysts in the orbit, or thrombosis of the cavernous sinus, or aneurism. But there are other forms, especially that occurring in exophthalmic goître, that require explanation. It has been held by some that the protrusion of the eyeball in Graves's disease is due to dilatation of the retro-bulbar vessels, while others have attributed the phenomenon to spasm of Müller's muscle; and these two theories formed the basis of the present investigation. Dogs were used for the experiments, and protrusion or retraction of the eyeball following the various manipulations and stimuli were recorded by means of a simple tracing apparatus on a revolving drum.

It was found that if the jugular veins and carotid arteries were separately clamped, definite changes in the position of the eye occurred. Closure of the external jugular vein on the same side as the eye which was under observation produced marked exophthalmos, which was greatly increased by closure of the external jugular on the other side. Clamping of the carotid arteries diminished the protrusion.

In another dog stimulation of the cervical sympathethic caused marked exophthalmos, and stimulation of the cervical sympathetic in the head of a decapitated dog produced as much exophthalmos as in the living dog. This and other experiments proved that the exophthalmos resulting from irritation of the sympathetic was not due to vascular changes. Further experiments showed that the thrusting forward of the eye was caused by contraction of the orbital muscle. As the authors point out, it does not follow that because contraction of the orbital muscle produces exophthalmos in dogs therefore it does so in man. The muscle is not so well developed in the human species, and experiments on two patients during operations in the region of the neck failed to produce exophthalmos by stimulation of the sympathetic. Neither were the authors able to find any spot in the course of the cervical sympathetic in dogs where stimulation caused protrusion of the eyeball without at the same time producing marked dilatation of the pupil. They state that their inquiry has not enabled them to draw certain conclusions as to the mechanism of the exophthalmos in Graves's disease. To us it appears that the experiments quoted are certainly in favour of vascular dilatation as the chief factor, though, of course, contraction of Müller's muscle may also play an active part.

[1] Med. News, Oct. 15.

